# PPARβ/δ Agonist Alleviates Diabetic Osteoporosis *via* Regulating M1/M2 Macrophage Polarization

**DOI:** 10.3389/fcell.2021.753194

**Published:** 2021-11-26

**Authors:** Miao Chen, Weimin Lin, Rui Ye, Jianru Yi, Zhihe Zhao

**Affiliations:** ^1^ State Key Laboratory of Oral Diseases and National Clinical Research Center for Oral Diseases, West China Hospital of Stomatology, Sichuan University, Chengdu, China; ^2^ Department of Orthodontics, West China Hospital of Stomatology, Sichuan University, Chengdu, China

**Keywords:** PPARβ/δ, diabetes mellitus, diabetic osteoporosis, macrophage polarization, inflammation

## Abstract

Diabetic osteoporosis is a common complication in diabetic patients, leading to increased fracture risk and impaired bone healing. As a member of the peroxisome proliferator-activated receptor (PPAR) family, PPARβ/δ agonist is suggested as a therapeutic target for the treatment of metabolic syndrome, and has been reported to positively regulate bone turnover by improving osteogenesis. However, its regulatory role in diabetic osteoporosis has not been reported yet. Here, we explored the therapeutic effects and potential mechanisms of PPARβ/δ agonist to the osteoporotic phenotypes of diabetic mice. Our results indicated that the osteoporotic phenotypes could be significantly ameliorated in diabetic mice by the administration of PPARβ/δ agonists. *In vitro* experiments suggested that PPARβ/δ agonist treatment could alleviate the abnormal increase of osteoclast activity in diabetic mice by rectifying high glucose-mediated macrophage dysfunction instead of directly inhibiting osteoclast differentiation. Mechanistically, *Angptl4* may act as a downstream target of PPARβ/δ to regulate macrophage polarization. In conclusion, our study demonstrates the potential of PPARβ/δ agonist as a therapeutic target for the treatment of osteoporosis and immune homeostasis disorder in diabetic patients.

## Introduction

Diabetes mellitus, a systemic metabolic disorder syndrome, is usually accompanied by hyperglycemia and chronic tissue and organ damage. In the last decade, the incidence of diabetes mellitus has increased rapidly worldwide with changes in diet and lifestyle (Unnikrishnan et al., 2016). The imbalance of immune homeostasis is one of the main characteristics of diabetic complications. Diabetes mellitus can lead to increased inflammatory infiltration, pro-inflammatory mediators including TNF-α, IL-1β, IL-6 and IL-18, resulting in aggravated inflammatory response (Graves and Kayal, 2008). ROS production in macrophages also increases under high glucose culture, resulting in abnormal macrophage polarization and disordered immune response (Rendra et al., 2019). Skeletal syndrome is an important complication of diabetic patients, including osteoporosis, increased fracture risk, and impaired bone healing properties. The inflammatory environment in diabetes mellitus suppressed the osteogenic differentiation and led to osteoblast apoptosis (Eller-Vainicher et al., 2020). Current evidence also suggested that diabetic status could stimulate osteoclast differentiation and bone resorption (Jiao et al., 2015). In diabetic patients, the circulating levels of tartrate-resistant acid phosphatase (TRAP) increased, which indicated the enhanced osteoclast activity (Suzuki et al., 2005). During the fracture healing process, diabetic rats showed increased number of osteoclasts and the up-regulated expression of inflammatory factors compared with healthy control (Kayal et al., 2007). Our previous study also found that hyperglycemia increased M1 macrophage polarization and osteoclast differentiation in diabetic rats, leading to the accelerated progression of periodontitis (Zhang et al., 2021).

Peroxisome proliferators-activated receptors (PPARs) belong to ligand-activated receptors in the nuclear hormone receptor family, which can bind to the PPAR response element (PPRE) of the target gene to regulate various intracellular processes (Khoo et al., 2013). As a member of PPARs, PPARβ/δ activation could alleviate osteoporosis by up-regulating Wnt signal pathway in osteoblasts (Scholtysek et al., 2013). PPARβ/δ knockout-mice were found to exhibit glucose intolerance and impaired bone formation (Fu et al., 2014). In addition, the activation of PPARβ/δ accelerated osteoblast differentiation and increased peroxisome number, which had the potential to improve oxidative stress overload (Qian et al., 2015). PPARβ/δ agonist could effectively reduce tissue damage caused by oxidative stress and promote wound repair (Barlaka et al., 2015; Jimenez et al., 2018). Loss of PPARβ/δ led to the failure of macrophages M2 polarization under IL-4 induction (Odegaard et al., 2008). However, its effects on diabetic osteoporosis and osteoclast differentiation have not been reported yet.

Therefore, we speculate that PPARβ/δ activation may be a possible therapeutic target for improving immune homeostasis imbalance and osteoporosis under diabetic condition. In this study, we explored the therapeutic effect of PPARβ/δ agonist GW501516 on osteoporotic phenotype in diabetic mice, and investigated the regulation effect of PPARβ/δ agonist on the oxidative stress and macrophages polarization in high glucose environment, as well as its regulatory effect on osteoclast differentiation.

## Materials and Methods

### Animal Model

The mice were purchased from Dashuo Company (Chengdu, China). Mice were housed in box cages, maintained on a 12 h light/12 h dark cycle, and fed a chow diet ad libitum (Sakai et al., 2019). The diabetes mellitus model of mice was constructed as described previously (Zhou X. et al., 2019). In brief, male C57BL/6J mice aged 8 weeks were fasted for 12 h and then injected intraperitoneally of 1% streptozotocin dissolved in saline solution with a dose of 50 mg/kg for 4 consecutive days. Tail vein blood was taken to test the fasting blood glucose level on the 1^st^ and 2^nd^ week post-injection. The mice with a fasting blood glucose higher than 16.7 mmol/L were regarded as successful diabetes model (Jiang et al., 2021). The mice were randomly divided into following three groups of eight mice each: 1) normal mice (Control); 2) normal mice treated with PPARβ/δ agonist GW501516 (Control + GW); 3) diabetes mellitus group (DM) and 4) diabetes mellitus group treated with PPARβ/δ agonist GW501516 (DM + GW).

Control + GW and DM + GW groups were injected with GW501516 (5 mg/kg/d, Sigma-Aldrich) dissolved in 0.1 ml dimethyl sulfoxide (DMSO) every other day (Mosti et al., 2014). Control group and DM group were injected with 0.1 ml DMSO every other day. The mice were sacrificed after 4 and 8 weeks of injection. The femurs were collected for subsequent histomorphological analysis.

### Micro–Computed Tomography Analysis

The femurs were dissected and fixed in 4% paraformaldehyde for 2 days and then stored in 70% ethanol at 4°C. Micro-computed tomography (μCT) analysis was performed (μCT50, SCANCO Medical) with a spatial resolution of 10 μm (55 kV, 114 mA, 500 ms integration time). The regions of interest (ROI) were defined as the trabecular bone and cortical bone at the distal femur (Bouxsein et al., 2010). Bone mineral density (BMD), bone volume/total volume (BV/TV), trabecular number (Tb.N), trabecular separation (Tb.Sp), trabecular thickness (Tb.Th), cortical bone thickness (Ct.Th), and cortical bone porosity (Ct.Po) were evaluated within the delimited ROI (Wang et al., 2020).

### Histomorphological Analysis

The mouse femurs were fixed and decalcified, and then embedded in paraffin. Sections of 4.5 μm were prepared. H&E staining (Solarbio, Beijing, China) was performed as the manufacturer’s instruction. The stained sections were observed using an inverted microscope (IX81, Olympus).

TRAP staining was performed according to the procedure described previously (Liu et al., 2016). TRAP staining solution (Sigma) was added to cover the tissue sections. After incubating at 37°C for 40 min, the sections were stained with 0.1% methyl green solution (Sigma) for 10 s. The stained sections were observed using an inverted microscope (IX81, Olympus).

### Bone Marrow-Derived Macrophage Culture

Eight-week-old C57BL/6J mice were sacrificed to separate the femur and tibia. The bone marrow was flashed into a petri dish and centrifuged. The supernatant was discarded, and the red blood cell lysate was added. The cells were seeded in a 10 cm diameter petri dish, cultured in DMEM medium with 10% fetal bovine serum (FBS), and supplied with 50 ng/ml m-CSF (R&D). After 4 days, the suspension cells were discarded and bone marrow-derived macrophages (BMDMs) were obtained for subsequent experiment.

DEME medium with 5.6 or 30 mM glucose were used for normal glucose (NG) or high glucose (HG) culture, and 5 μM GW501516 was added to HG + GW group. After 3 days of culture, different macrophage culture supernatants were obtained. To avoid the inhibition of osteoclast differentiation by high glucose concentration, DMEM medium without glucose was used to mix with the culture supernatant. Osteoclast-induced BMDMs are divided into three groups: NG Supernatant group, HG Supernatant group, and HG + GW Supernatant group.

### Osteoclast Induction

For osteoclast induction, BMDMs (4×10^4^ cells/well) were seeded in 24-well plates. 50 ng/ml RANKL (R&D) was added to the BMDM culture medium, and the medium was changed every 2 days. After 3 days of culture, mature osteoclasts could be observed and subsequent experiments were performed.

TRAP staining (Sigma-Aldrich) was performed according to the previously described procedure (Liu et al., 2016). The cells were fixed with 4% paraformaldehyde for 10 min, added with TRAP staining solution, and incubated at 37°C for 40 min. TRAP-positive cells containing three or more nuclei were considered osteoclasts.

### Reverse Transcription Polymerase Chain Reaction

The total RNA of BMDMs and osteoclasts was extracted using Trizol reagent (Invitrogen, Carlsbad, CA, United States), and then reversely transcribed to obtain stable cDNA using PrimeScript™ RT reagent Kit with gDNA Eraser (TaKaRa Bio, Otsu, Japan). The RT-PCR was performed using SYBR Premix Ex Taq II (TaKaRa Bio) in Quant Studio™ three real-time fluorescent PCR instrument (ThermoFisher Scientific, China). Glyceraldehyde 3-phosphate dehydrogenase (*Gapdh*) was used as an internal reference to normalize the gene expression (Wang et al., 2020). The result was calculated using the 2^−ΔΔCt^ method and expressed as a multiple change relative to *Gapdh*. The primer sequences are summarized in Supplementary Table S1.

### ROS Detection

The detection of ROS levels in BMDMs was carried out in accordance with the recommended protocol (Pang et al., 2019). In brief, BMDMs (2×10^5^ cells/well) were seeded in 6-well plates under NG, HG, or HG + GW culture. Then, the ROS fluorescent probe DCFH-DA (Beyotime Biotechnology) was diluted with serum-free DMEM medium at a ratio of 1:1,000. After culturing for 72 h, the culture medium was removed and then DMEM medium containing DCFH-DA was added. After incubation at 37°C for 20 min, the cells were washed three times with serum-free DMEM medium without phenol red and digested by trypsin. The fluorescence intensity was analyzed on a flow cytometer (ThermoFisher).

### Flow Cytometry

For flow cytometry analysis, the mouse femur was cut into pieces, digested in 1 mg/ml collagenase I, 1 mg/ml dispase II at 37°C for 30 min, centrifuged. After lysis of red blood cells on ice, the samples were passed through a 70-μm filter, centrifuged, and ready for staining. Anti-mouse CD45, anti-mouse CD11b, anti-mouse CD86, and anti-mouse CD206 were purchased from BD Biosciences, and the permeabilization/fixation kit was purchased from eBioscience. All staining processes were performed in 100 μl PBS. The cells were stained on ice for 30 min and then analyzed by flow cytometry (BD Biosciences).

### ELISA

The expression levels of TNF-α and IL-1β were measured by mouse ELISA kits (Cusabio) according to the recommended protocol. Briefly, 100 μl of culture supernatant was added to a 96-well plate with high binding capacity and incubated for 2 h, and the absorbance was measured with a microplate reader. Serum concentration of PINP, CTX and ANGPTL4 were measured using ELISA kits (CUSABIO). Mice were fasted for 4 h and then we collected the blood samples by puncturing the cheek pouch and allowed the blood to coagulate on ice for 1 h before centrifugation to obtain the serum.

### Gene Knockdown

siRNA sequence for *Angptl4* and *Ppard* were designed and synthesized by Sangon Biotech (Shanghai, China). BMDMs were transfected with Lipofectamine® RNAimax (Invitrogen) in serum-free DMEM medium followed by the manufacturer’s instructions. In all experiments using siRNA, control siRNA and Lipofectamine® RNAimax were added to other group to eliminate other potential influence.

### Immunofluorescence Staining

For immunofluorescence staining, sodium citrate solution was used for antigen retrieval. After blocking by 5% BSA at 37°C for 1 h, primary antibodies (anti-CD86, anti-CD206, Abcam) were incubated overnight at 4°C. Then the fluorescent secondary antibodies (Abcam) were incubated for 1 h at room temperature. DAPI was used to mark the nucleus with 15 min of staining. The stained sections were observed under a fluorescent microscope (Olympus BX53).

### Chromatin Immunoprecipitation

Chromatin immunoprecipitation (ChIP) assay was performed according to the manufacturer’s instructions with EZ-Zyme Chromatin Preparation Kit (Millipore) and Magna Chip HiSens (Millipore). Rabbit IgG (Sigma) was used as the control, and antibody against PPARβ/δ (Santa Cruz) was set as the experimental group. The DNA-protein complex was dissociated, PCR primers were designed to detect the target area, and then the pull-down DNA and input DNA were tested by PCR analysis with the primers flanking the PPRE region of *Angptl4*.

For ChIP-seq analysis, we downloaded the ChIP-seq data of PPARβ/δ in GEO database, with the accession number GSE50144 (Inoue et al., 2014). The binding peak of PPARβ/δ in *ANGPTL4* gene segment was visualized *via* integrative genomics viewer (IGV) software.

### Statistical Analysis

All quantified data were expressed as mean ± standard deviation (SD). Statistical differences were performed *via* unpaired two-tailed Student’s *t* test for comparison between two groups and by one-way or two-way analysis of variance (ANOVA) followed by the Tukey’s post hoc test for multiple comparisons. *p* values < 0.05 were considered to be statistically significant.

## Results

### PPARβ/δ Agonist Alleviates the Osteoporotic Phenotypes of Diabetic Mice *in Vivo*

**FIGURE 1:**
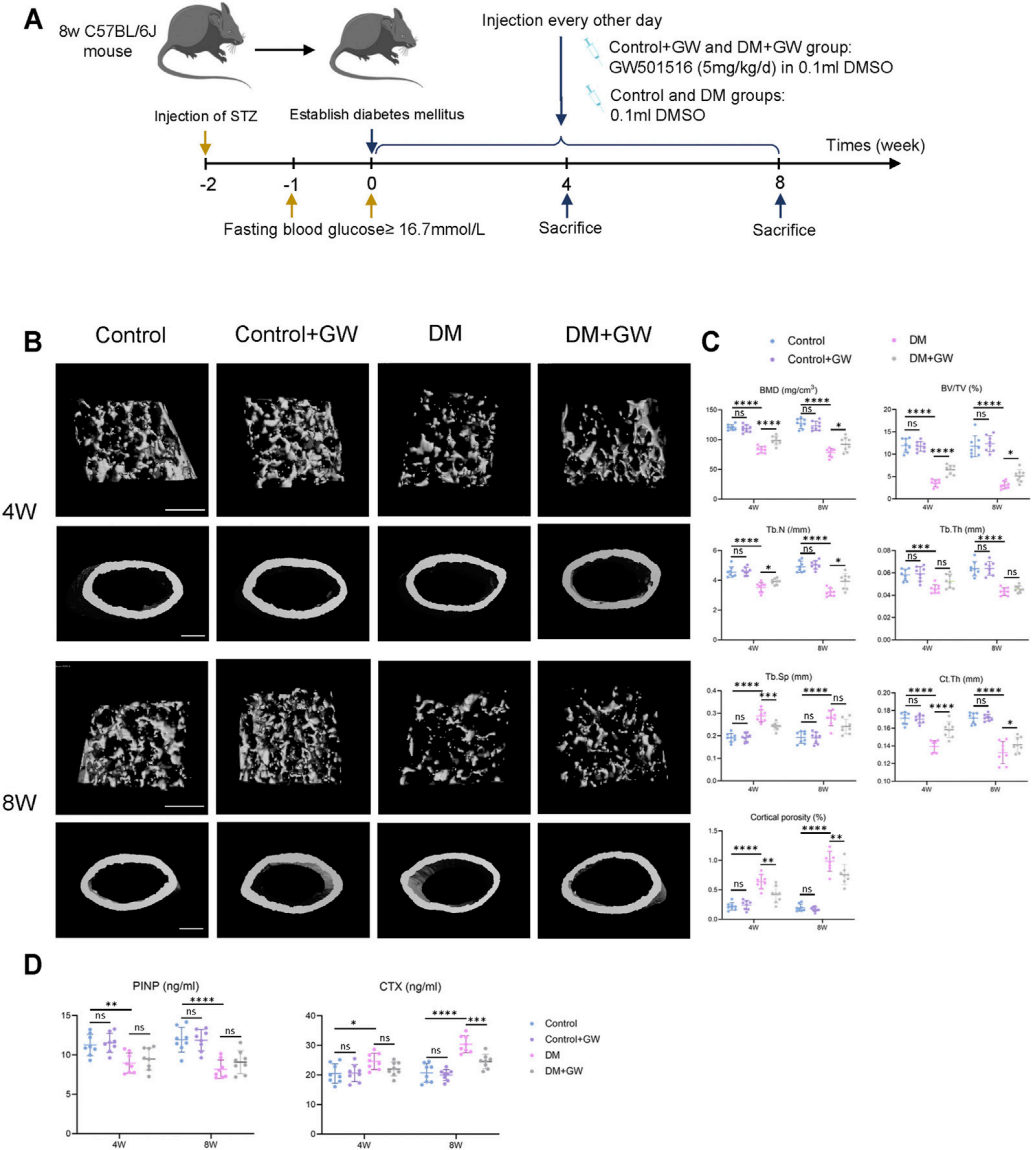
PPARβ/δ agonist alleviates the osteoporosis phenotype in diabetic mice. **(A)** Schematic illustration of experimental design for PPARβ/δ agonist treatment. **(B)** Representative images of μCT of distal femoral tissue at the 4^th^ and 8^th^ week. Scale bar = 500 µm. **(C)** Quantitative μCT analyses of bone mineral density (BMD), bone volume/total volume (BV/TV), trabecular number (Tb.N), trabecular separation (Tb.Sp), trabecular thickness (Tb.Th), cortical bone thickness (Ct.Th), and cortical bone porosity (Ct.Po) of distal femoral tissue (*n* = 8). **(D)** Serum levels of PINP and CTX in each group. Data were expressed as mean ± SD. The *p* values were calculated by two-way ANOVA. (ns, not statistically significant, *p < 0.05, **p < 0.01, ***p < 0.001, ****p < 0.0001).

**FIGURE 2:**
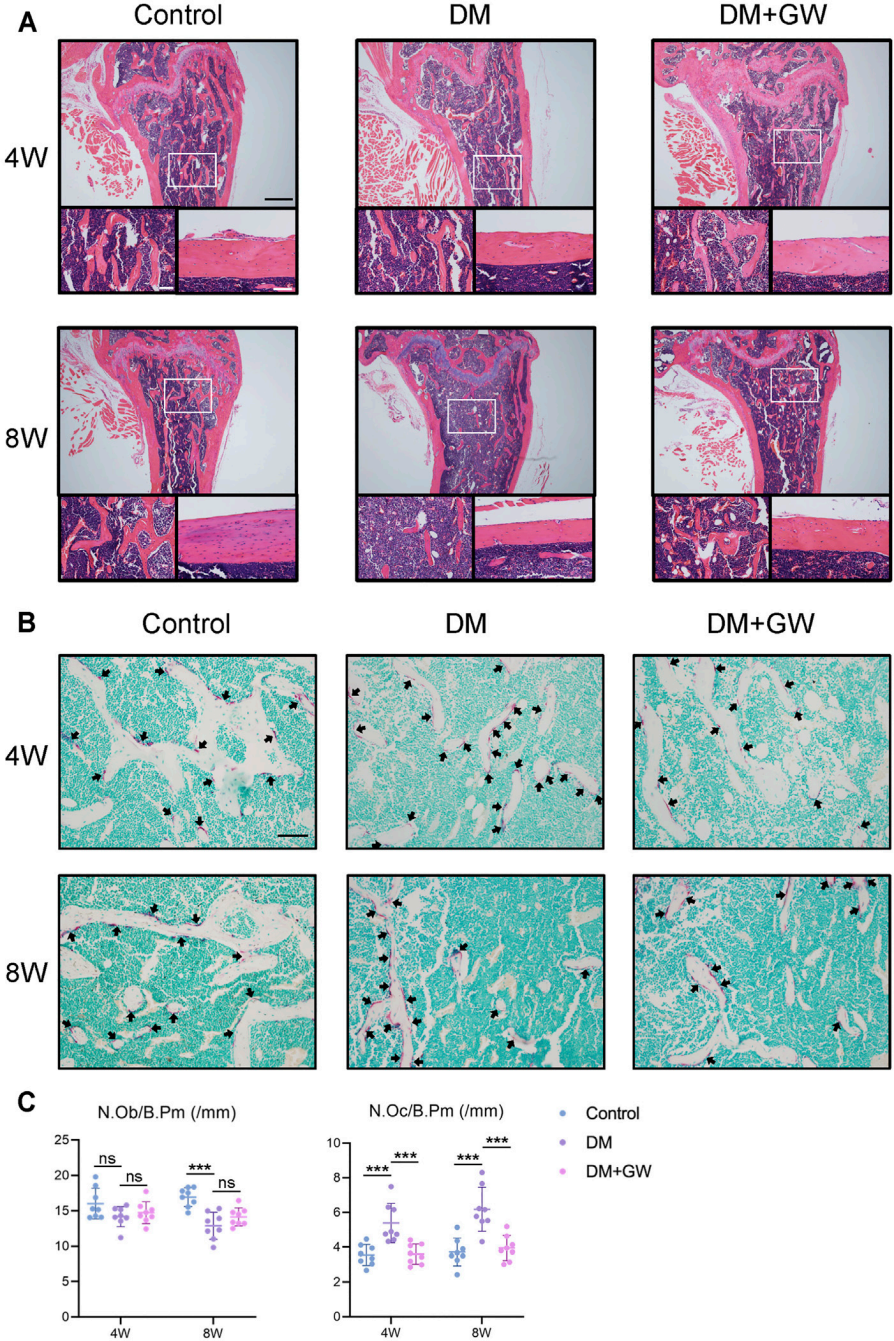
PPARβ/δ agonist reduces the bone loss and osteoclast activation in diabetic mice. **(A)** Representative images of H&E staining of distal femoral tissue sections. Scale bar = 500 µm (top), scale bar = 100 µm (bottom). **(B)** Representative images of TRAP staining of distal femoral tissue sections. TRAP positive cells were indicated by arrows. Scale bar = 100 µm. **(C)** Statistics for the number of osteoblasts and osteoclasts. Data were expressed as mean ± SD. The *p* values were calculated by one-way ANOVA. (ns, not statistically significant, ***p < 0.001). N. Ob/B.Pm = osteoblast number/bone perimeter, N. Oc/B.Pm = osteoclast number/bone perimeter.

First, we explored the effect of PPARβ/δ agonist on the osteoporotic phenotypes of diabetic mice (Figure 1A). Through μCT analysis of the distal femur, we found that both trabecular bone and cortical bone were affected by the diabetic condition and manifested as bone loss. PPARβ/δ agonist could partially relieve the diabetic osteoporosis. In addition, PPARβ/δ agonist treatment had no significant influence on bone phenotype in healthy control mice (Figures 1B,C). H&E staining results showed the number and thickness of trabecular bone and the thickness of cortical bone decreased in DM group, while in the DM + GW group, the osteoporotic phenotype was restored (Figure 2A). Through TRAP staining, we found that osteoclast activity in DM group was significantly increased, and PPARβ/δ agonist treatment could reduce the activation of osteoclasts under diabetic condition (Figures 2B,C). Also, bone resorption marker CTX decreased after PPARβ/δ agonist treatment (Figure 1D). Overall, we found that at the 4^th^ week and 8^th^ week, the bone mass of the DM group was significantly lower than that of the control group, and PPARβ/δ agonist alleviated the diabetic bone loss.

### PPARβ/δ Agonist has no Direct Effect on Osteoclast Differentiation *in Vitro*

**FIGURE 3:**
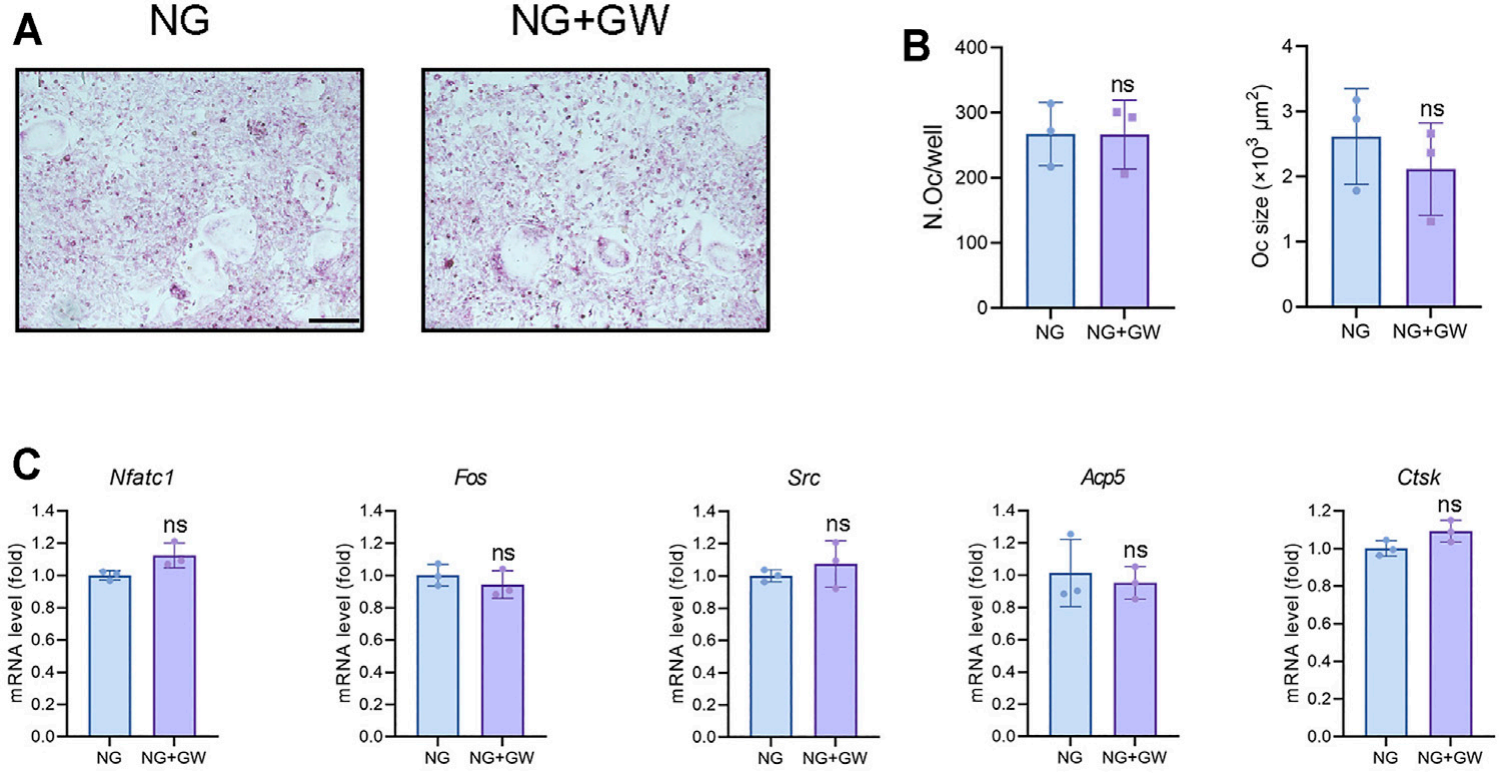
PPARβ/δ agonist cannot directly regulate osteoclast differentiation *in vitro*. **(A)** TRAP staining for osteoclast differentiation before and after PPARβ/δ agonist treatment. Scale bar = 100 μm. **(B)** Statistics on the number and size of osteoclasts. **(C)** RT-PCR results of the expressions of the osteoclastogenesis-related genes before and after PPARβ/δ agonist treatment. Data were expressed as mean ± SD. The *p* values were calculatedby two-tailed Student’s t test. (ns, not statistically significant). N. Oc = number of osteoclasts.

Seizing the evidence that PPARβ/δ agonist alleviated the diabetic osteoporosis phenotypes and reduced osteoclast activity *in vivo*, we speculated that PPARβ/δ might directly inhibit osteoclast differentiation. Considering that high glucose environment inhibited osteoclast differentiation *in vitro* (Wittrant et al., 2008), we explored the regulatory effect of PPARβ/δ agonist on osteoclast differentiation under normal glucose culture. However, PPARβ/δ agonist exhibited no effect on osteoclast differentiation *in vitro* (Figures 3A,B). RT-PCR results showed there is no significant difference in osteoclastogenesis-related genes before and after PPARβ/δ agonist treatment (Figure 3C).

### PPARβ/δ Agonist Improves High Glucose-Mediated Macrophage Inflammation

**FIGURE 4:**
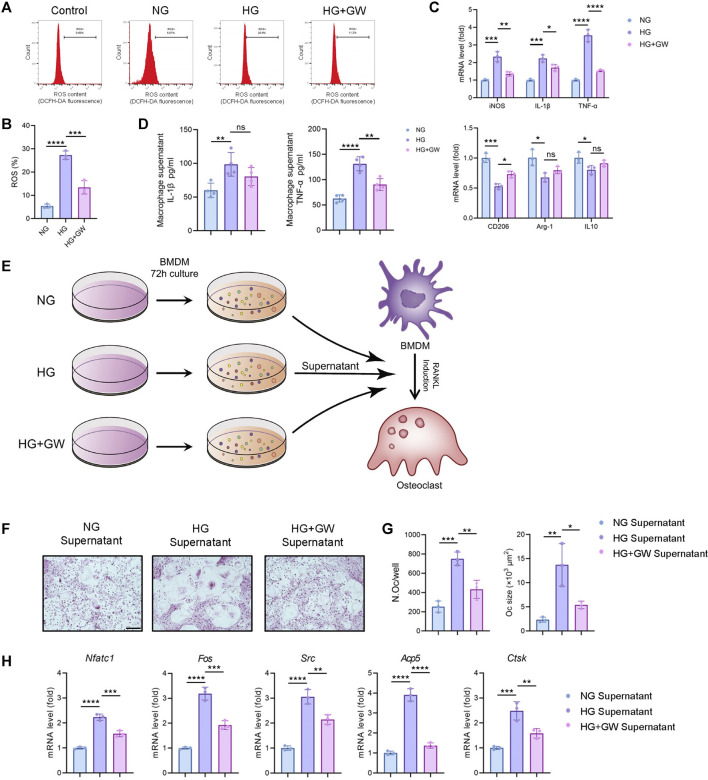
PPARβ/δ agonist alleviates high glucose-induced macrophage inflammation and supernatant-mediated osteoclast differentiation. **(A)** Flow cytometry analysis of ROS levels detected by fluorescent probe in different groups. **(B)** Statistics for the ROS levels. **(C)** RT-PCR results regarding the expression levels of macrophage polarization-related genes in different groups. **(D)** Expression level of IL-1β and TNF-α detected by ELISA. **(E)** Schematic illustration of experimental design for regulating osteoclast differentiation through macrophage culture supernatant. **(F)** Representative images of TRAP staining for osteoclast differentiation in different groups. Scale bar = 100 μm. **(G)** Statistics for the number and size of osteoclasts. **(H)** RT-PCR results of the expressions of the osteoclastogenesis-related genes in different groups. Data were expressed as mean ± SD. The *p* values were calculated by one-way ANOVA. (ns, not statistically significant, *p < 0.05, **p < 0.01, ***p < 0.001, ****p < 0.0001).

Given that immune homeostasis imbalance was an important factor leading to diabetic complications, we explored the effect of PPARβ/δ agonist one the ROS level and pro-inflammatory polarization of macrophages induced by high glucose. The results showed that PPARβ/δ agonist effectively reduced the high glucose-induced ROS production (Figures 4A,B) and the expression of M1 signature genes (iNOS, IL-1β, and TNF-α), while improved the expression of M2 signature genes (CD206, Arg-1, and IL-10) (Figure 4C).

Our previous studies proved that the macrophage polarization could affect osteoclast differentiation (Zhang et al., 2021). Therefore, we supposed that the reduced inflammatory phenotypes of macrophage might be the key mechanism for PPARβ/δ agonist to alleviate diabetic osteoclast activity. After adding macrophage culture supernatant of NG, HG, and HG + GW groups, the differentiation levels of osteoclasts were different (Figure 4E). The macrophage culture supernatant from HG group could significantly increase both the number and size of osteoclasts, while supernatant from HG + GW group reversed the stimulating effect on osteoclast differentiation (Figures 4F,G). The results of RT-PCR showed that the macrophage culture supernatant from HG group could significantly increase the expression of osteoclastogenesis-related genes, which were partially reduced by HG + GW Supernatant group (Figure 4H). To clarify which factor in the culture supernatant affected the osteoclast differentiation, we extracted the macrophage culture supernatant and measured the protein levels of IL-1β and TNF-α by ELISA. We found that high-glucose culture increased the expression level of IL-1β and TNF-α, and PPARβ/δ agonist treatment significantly decreased IL-1β and TNF-α level, and the reduction of TNF-α was the most significant (Figure 4D). In addition, after neutralizing antibodies treatment, we found that TNF-α blockade had the most obvious effect on inhibiting osteoclast differentiation, and IL-1β blockade also inhibited the osteoclast differentiation (Supplementary Figure S1). We speculate that TNF-α is the most important inflammatory factor that stimulates osteoclast differentiation under high glucose, and PPARβ/δ agonist treatment can reduce osteoclast differentiation by reducing the level of TNF-α.

### Abnormal M1/M2 Polarization is Restored by PPARβ/δ Agonist in Diabetic Mice

**FIGURE 5:**
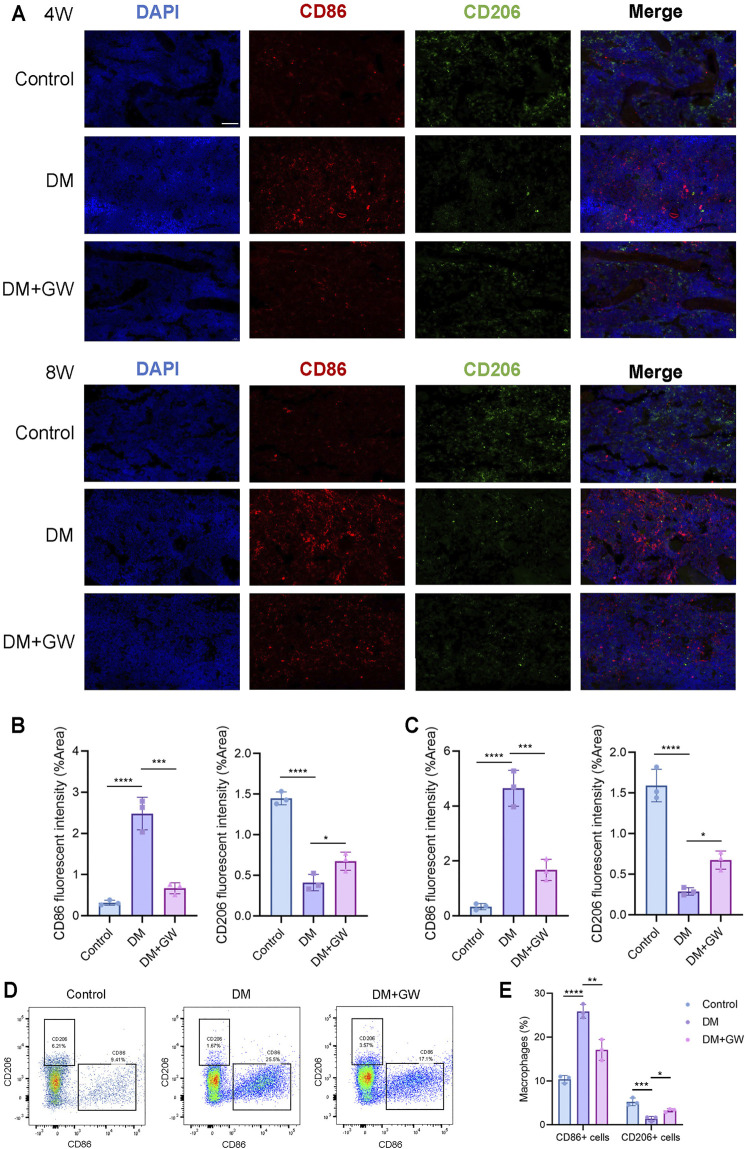
PPARβ/δ agonist improves the abnormal macrophage polarization in skeletal system of diabetic mice. **(A)** Representative images of immunofluorescence staining of the M1/M2 macrophage polarization at the 4^th^ and 8^th^ week is in control, DM and DM + GW group. Red-CD86, Green-CD206, Blue-DAPI. Scale bar = 50 μm. **(B)** Quantitative analysis of CD86 and CD206 fluorescence intensity at the 4^th^ week. **(C)** Quantitative analysis of CD86 and CD206 fluorescence intensity at the 8^th^ week. **(D)** Flow cytometry analysis of CD86^+^ and CD206^+^ macrophage proportion in each group. **(E)** Statistics for CD86^+^ and CD206^+^ macrophage proportion. Data were expressed as mean ± SD. The *p* values were calculated by one-way ANOVA. (*p < 0.05, **p < 0.01, ***p < 0.001, ****p < 0.0001).

To validate that PPARβ/δ agonist could reduce the inflammatory phenotypes of macrophage *in vivo*, immunofluorescence staining (Figures 5A–C) and flow cytometry analysis (Figures 5D,E) of bone marrow tissue were performed to detect the expression of CD86 (M1 marker) and CD206 (M2 marker). The CD86^+^ cells in the DM group significantly increased compared to control group, while the number of CD206^+^ cells decreased, indicating that M1 polarization of macrophages increased while the M2 polarization decreased in DM group. PPARβ/δ agonist reduced the proportion of CD86^+^ cells and enhanced the proportion of CD206^+^ cells, indicating that PPARβ/δ agonist treatment *in vivo* could effectively rectify the macrophage dysfunction in skeletal system under diabetic condition.

### PPARβ/δ Agonist Treatment Restored the M1/M2 Polarization *via* Up-Regulating *Angptl4*

**FIGURE 6:**
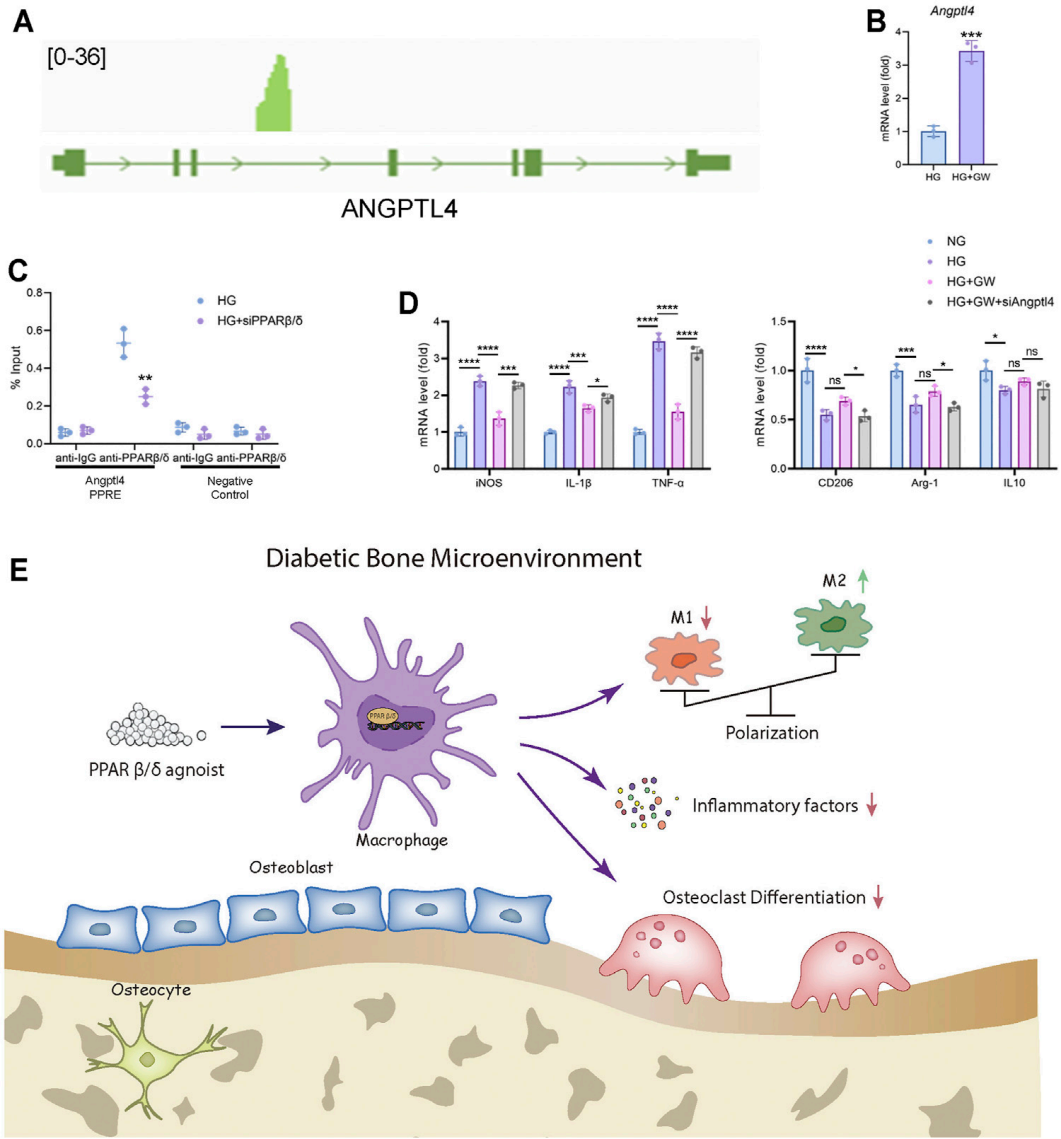
*Angptl4* is a downstream target of PPARβ/δ agonist to exert an anti-inflammatory effect in macrophage. **(A)** ChIP-seq analysis of the binding peak of PPARβ/δ in *Angptl4* gene segment. **(B)** RT-PCR result of the expression level of *Angptl4* after PPARβ/δ agonist treatment in macrophage. **(C)** ChIP-PCR validation of the binding of PPARβ/δ to the PPRE region of *Angptl4*. **(D)** RT-PCR results of PPARβ/δ knockdown on the expression of macrophage polarization-related genes. **(E)** The mechanism diagram of PPARβ/δ agonist alleviating diabetic osteoporosis. Data were expressed as mean ± SD. The *p* values were calculated by two-tailed Student’s t test or one-way ANOVA. (ns, not statistically significant, *p < 0.05, **p < 0.01, ***p < 0.001, ****p < 0.0001).

After determining the regulatory effect of PPARβ/δ agonists on macrophage polarization *in vivo* and *in vitro*, we hope to explore the regulatory mechanism and downstream signaling pathways of PPARβ/δ agonist. As an important transcription factor, PPARβ/δ had been reported to bind to the PPRE region of genes and activate gene transcription. By analyzing ChIP-seq data in GEO database, we found that there is a binding peak at the intronic region of *Angptl4*, which is reported to coincide with the PPRE region (Inoue et al., 2014) (Figure 6A). Based on the above evidence, we speculated that *Angptl4* could serve as a potential downstream gene of PPARβ/δ.

In mice BMDM, PPARβ/δ agonist treatment also up-regulated the expression level of *Angptl4* (Figure 6B). Through ChIP assay, we proved that PPARβ/δ had a binding site in the gene segment of *Angptl4* in BMDM (Figure 6C). Knockdown of *Angptl4* by siRNA in BMDM eliminated the protective effect of PPARβ/δ agonist on the inflammatory phenotypes of macrophages under high-glucose culture (Figure 6D). We evaluated the expression level of ANGPTL4 in serum of diabetic mice by ELISA test and verified ANGPTL4 expression in bone marrow tissue through immunohistochemical staining. We found that after PPARβ/δ agonist treatment, ANGPTL4 was significantly up-regulated (Supplementary Figure S2). However, as for the effect of secreted ANGPTL4, blockade of ANGPTL4 by neutralizing antibody in culture supernatant did not significantly affect osteoclast differentiation, which suggested that the regulation of osteoclast differentiation by PPARβ/δ agonist seemed to be independent of secreted ANGPTL4 (Supplementary Figure S3). Therefore, we believed that *Angptl4* could serve as an important downstream gene of PPARβ/δ to regulate macrophage polarization (Figure 6E).

## Discussion

Diabetes mellitus disturbs bone metabolism, leading to diabetic osteoporosis and increased fracture risk (Schwartz, 2017). Successful glycemic control can reduce the incidence of diabetes osteoporosis (Paschou et al., 2017). Agonists of the PPAR family were developed to treat metabolic syndrome. However, for the skeletal system, PPAR α and PPAR γ agonists had been shown to exert a negative impact on osteoblast differentiation and promote adipogenesis (Hauner, 2002; Patel et al., 2014). Different from the other two members, PPARβ/δ agonist was believed to be beneficial to the skeletal system and could promote osteogenic differentiation (Scholtysek et al., 2013). Therefore, PPARβ/δ agonist seemed to be a promising target for protecting skeletal health.

Abnormal activation of the immune system is one of the main manifestations of diabetes mellitus, leading to the inflammatory damage. Increased polarization of M1 macrophages under diabetic status enhanced the secretion of inflammatory factors such as TNF-α, which further promoted the differentiation of osteoclasts (Jiao et al., 2015). Treatment with LPS-induced M1 macrophage culture supernatant stimulated the osteoclastogenesis (Zhang et al., 2021). PPARβ/δ agonist has been found to reduce the expression of inflammatory factors such as IL-1β and IL-6 by inhibiting NF-κB signaling (Rival et al., 2002; Bishop-Bailey and Bystrom, 2009; Coll et al., 2010), and promote the recruitment of M2 macrophages to accelerate the injury repair process (Tobita et al., 2020). However, its role in diabetic osteoporosis has not been reported yet. In the current study, we proved that PPARβ/δ agonist could alleviate the oxidative stress response and inflammatory products of macrophages induced by high glucose *in vitro*. Moreover, PPARβ/δ agonist could also reduce the stimulating effect of high glucose-induced macrophage culture supernatant on osteoclast differentiation. Through immunofluorescence staining and flow cytometry, we confirmed that PPARβ/δ agonist could improve the diabetes-mediated imbalance of macrophage polarization *in vivo*. Based on the above evidence, we believe that PPARβ/δ agonist can alleviate osteoclast differentiation and bone loss by reducing the M1 macrophage polarization under diabetic condition. In addition to inhibiting osteoclast differentiation, PPARβ/δ agonists could also promote bone formation (Scholtysek et al., 2013), but the bone resorption markers decreased more obviously after PPARβ/δ agonist treatment. Therefore, the inhibition of bone resorption and osteoclast differentiation might play a major role in PPARβ/δ agonist-mediated bone loss protection.

PPARβ/δ, a transcription factor, could regulate the transcription of downstream genes *via* a ligand-activated manner (Liu et al., 2018). Among them, *Angptl4* was considered to be an important downstream target of PPARβ/δ transcriptional regulation (Legrand et al., 2019). *Angptl4* had been reported to affect immune response and macrophage polarization (Cho et al., 2019). *Angptl4* knockout mice showed a large number of macrophage infiltration and high expression of TNF-α, IL-1β and other inflammatory factors (Oteng et al., 2019). During *in vitro* culture, the level of TNF-α in the supernatant of *Angptl4* −/− macrophages was higher than that of wild-type macrophages (Ding et al., 2020). By administering recombinant human ANGPTL4 protein *in vitro*, Zhou et al. proved that ANGPTL4 could promote the M2 macrophage polarization and facilitate cardiac repair (Zhou et al., 2020). Hence, we supposed that *Angptl4* could act as a downstream gene of PPARβ/δ in macrophages to exert an anti-inflammatory effect. PPARβ/δ agonist increased the expression of *Angptl4* and ChIP assay proved that PPARβ/δ could bind to the *Angptl4* gene segment. Knockdown of *Angptl4* could eliminate the improvement to abnormal polarization of macrophages by PPARβ/δ agonist. Therefore, activating the transcription of *Angptl4* might be a downstream target for PPARβ/δ agonist to regulate macrophage polarization.

In conclusion, our study proves that PPARβ/δ agonist can effectively reduce the osteoporotic phenotype of diabetic mice. PPARβ/δ agonist ameliorates macrophage polarization imbalance and osteoclast abnormal activation under diabetic condition. *Angptl4* is considered as a key downstream factor for the biological effects of PPARβ/δ to regulate macrophage polarization. However, it should be noted that GW501516 has been reported to have potential pro-oncogenic effect, which affected its clinical application (Zhou D. et al., 2019; Liu et al., 2019). We believe that PPARβ/δ agonists without pro-oncogenic effect could be developed, and PPAR agonist treatment may serve as a promising therapeutic target for diabetic osteoporosis.

## Data Availability

The original contributions presented in the study are included in the article/Supplementary Material, further inquiries can be directed to the corresponding authors.
