# Supplementary material for: PPARβ/δ Agonist Alleviates Diabetic Osteoporosis via Regulating M1/M2 Macrophage Polarization
Source: Front Cell Dev Biol. 2021 Nov 26;9:753194. doi: 10.3389/fcell.2021.753194 (PMC8661472; doi:10.3389/fcell.2021.753194)
Supplement: Supplementary file 1 [file Table1.docx]

Supplementary Table 1. The primer sequences for RT-PCR.

| qRT-PCR (mouse) | |  |
| --- | --- | --- |
| Genes | **Primers** | **Sequences (5*’*‐3*’*)** |
| *Gapdh* | Forward | ACTGAGGACCAGGTTGTC |
|  | Reverse | TGCTGTAGCCGTATTCATTG |
| *Nfatc1* | Forward | GGAGAGTCCGAGAATCGAGAT |
|  | Reverse | TTGCAGCTAGGAAGTACGTCT |
| *Fos* | Forward | CGGGTTTCAACGCCGACTA |
|  | Reverse | TTGGCACTAGAGACGGACAGA |
| *Src* | Forward | GAACCCGAGAGGGACCTTC |
|  | Reverse | GAGGCAGTAGGCACCTTTTGT |
| *Acp5* | Forward | CACTCCCACCCTGAGATTTGT |
|  | Reverse | CATCGTCTGCACGGTTCTG |
| *Ctsk* | Forward | GAAGAAGACTCACCAGAAGCAG |
|  | Reverse | TCCAGGTTATGGGCAGAGATT |
| *iNOS*  *(Nos2)* | Forward | CAAGCACCTTGGAAGAGGAG |
|  | Reverse | AAGGCCAAACACAGCATACC |
| *IL-1β*  *(Il1b)* | Forward | CAAATCTCGCAGCAGCACA |
|  | Reverse | TCATGTCCTCATCCTGGAAGG |
| *TNF-α*  *(Tnf)* | Forward | AGGGTCTGGGCCATAGAACT |
|  | Reverse | CCACCACGCTCTTCTGTCTAC |
| *CD206*  *(Mrc1)* | Forward | CAAGGAAGGTTGGCATTTGT |
|  | Reverse | CCTTTCAGTCCTTTGCAAGC |
| *ARG-1*  *(Arg1)* | Forward | TCACCTGAGCTTTGATGTCG |
|  | Reverse | CTGAAAGGAGCCCTGTCTTG |
| *IL-10*  *(Il10)* | Forward | CCAAGCCTTATCGGAAATGA |
|  | Reverse | TTTTCACAGGGGAGAAATCG |
| *Angptl4* | Forward | GGGACTGCCAGGAACTCTTC |
|  | Reverse | GAAGTCCACAGAGCCGTTCA |
