# Supplementary material for: PPARβ/δ Agonist Alleviates Diabetic Osteoporosis via Regulating M1/M2 Macrophage Polarization
Source: Front Cell Dev Biol. 2021 Nov 26;9:753194. doi: 10.3389/fcell.2021.753194 (PMC8661472; doi:10.3389/fcell.2021.753194)
Supplement: Supplementary file 2 [file Image2.pdf]

Supplementary Figure 2

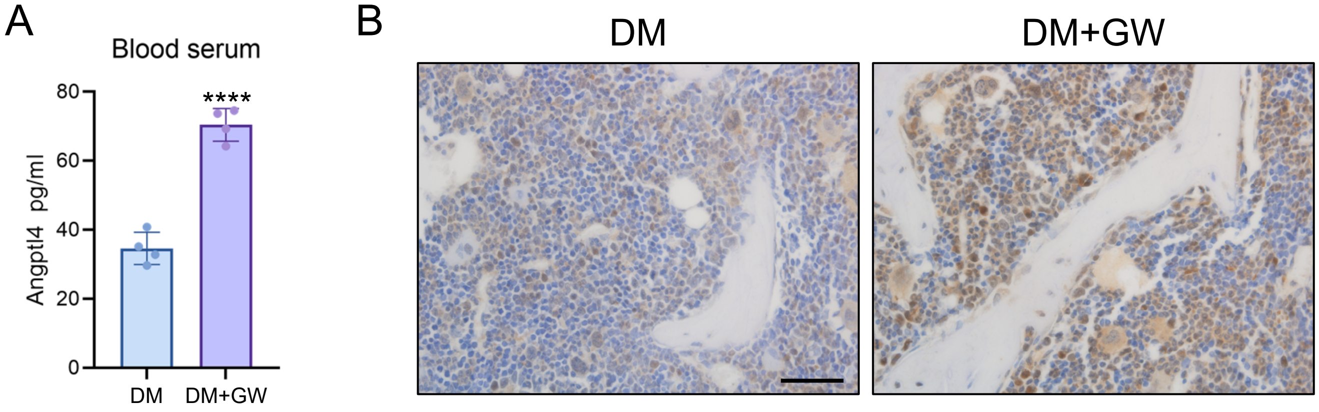

Supplementary Figure 2. The expression level of ANGPTL4 in serum and bone marrow tissue of diabetic mice through (A) ELISA test and (B) immunohistochemical staining. Scale bar = 50μm. Data were expressed as mean ± SD. The *p* values were calculated by two-tailed Student's t test. (****p <0.0001).
