# Supplementary material for: PPARβ/δ Agonist Alleviates Diabetic Osteoporosis via Regulating M1/M2 Macrophage Polarization
Source: Front Cell Dev Biol. 2021 Nov 26;9:753194. doi: 10.3389/fcell.2021.753194 (PMC8661472; doi:10.3389/fcell.2021.753194)
Supplement: Supplementary file 3 [file Image3.pdf]

Supplementary Figure 3

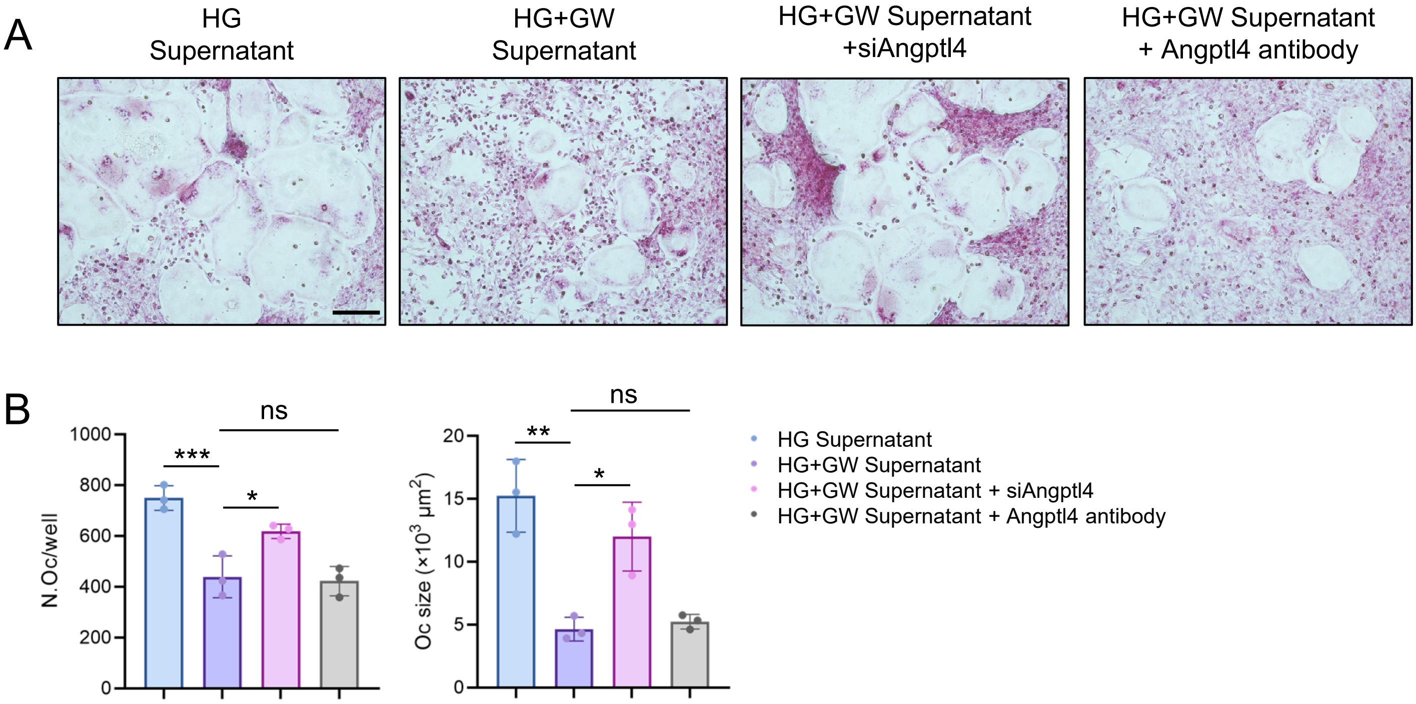

Supplementary Figure 3. The effect of *Angptl4* knockdown or secreted ANGPLT4 on the osteoclast differentiation *in vitro*. (A) TRAP staining for osteoclast differentiation. Scale bar = 100μm. (B) Statistics on the number and size of osteoclasts. Data were expressed as mean ± SD. The p values were calculated by one-way ANOVA. (ns, not statistically significant, *$p < 0.05$, **$p < 0.01$, ***$p < 0.001$).
