# Supplementary material for: PPARβ/δ Agonist Alleviates Diabetic Osteoporosis via Regulating M1/M2 Macrophage Polarization
Source: Front Cell Dev Biol. 2021 Nov 26;9:753194. doi: 10.3389/fcell.2021.753194 (PMC8661472; doi:10.3389/fcell.2021.753194)
Supplement: Supplementary file 4 [file Image1.pdf]

Supplementary Figure 1

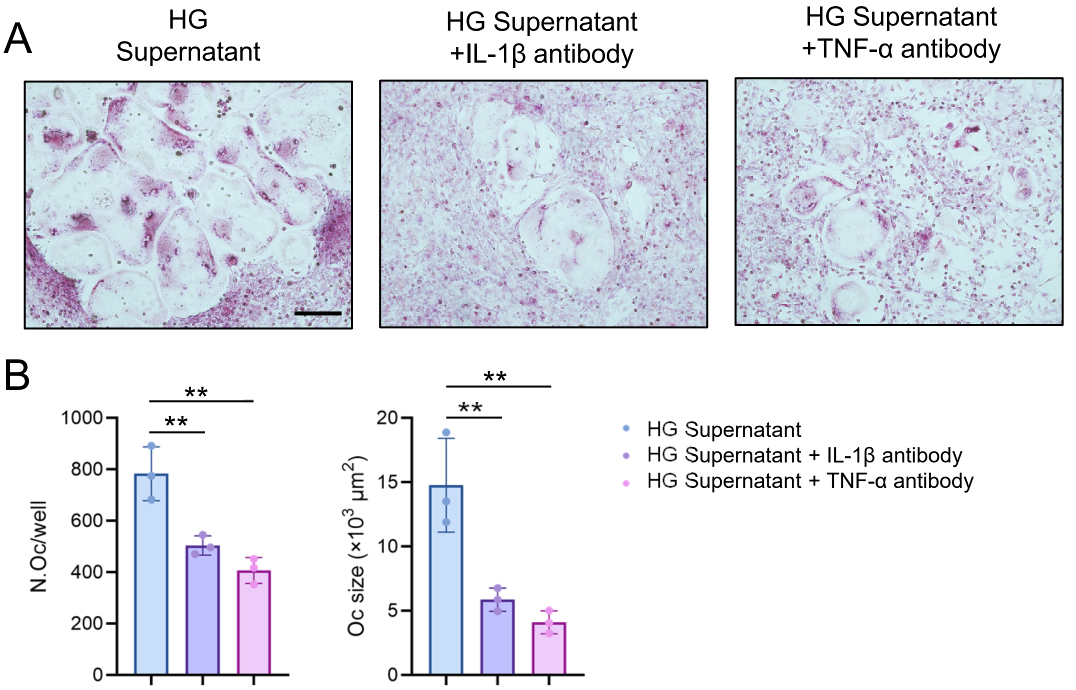

Supplementary Figure 1. TNF-α and IL-1β blockades inhibited the osteoclast differentiation *in vitro*. (A) TRAP staining for osteoclast differentiation after neutralizing antibodies treatment. Scale bar = 100μm. (B) Statistics on the number and size of osteoclasts. Data were expressed as mean ± SD. The *p* values were calculated by one-way ANOVA. (**$p < 0.01$).
